# Age and Sex Moderate the Effects of Sleep Quality on Resting-State Functional Connectivity in the Salience and Default Mode Network

**DOI:** 10.21203/rs.3.rs-7066049/v1

**Published:** 2025-07-14

**Authors:** Selene Tan, Sepehr Gourabi, Matthew R. Cribbet, Jeanne M. Cundiff, Ian M. McDonough

**Affiliations:** The University of Alabama; Binghamton University; The University of Alabama; The University of Alabama; Binghamton University

**Keywords:** fMRI, PSQI, sex, aging, functional connectivity, sleep

## Abstract

Aging often coincides with declining sleep quality, which can contribute to cognitive decline and increased dementia risk. Neuroimaging offers valuable insights into how poor sleep may affect brain health before cognitive or behavioral changes appear. Given the different prevalence rates of dementia between sexes, sleep disturbances might uniquely impact females relative to males. The goal of this study was to investigate how age and sex moderate the impact of sleep quality on resting-state functional connectivity. Based on the extant literature, we predicted that sleep quality would significantly impact connectivity in the default mode network, salience network, and the amygdala. Adults (N = 95), aged 20–74, completed the Pittsburgh Sleep Quality Index and underwent two 5-min sessions of resting-state MRI. Three-way interactions between age, sex, and sleep quality were found between the default mode network and the left superior parietal lobule. A two-way interaction between age and sleep quality was found between the salience network and right precentral/postcentral gyrus. No relationships were found using the amygdala as a seed region. Furthermore, sleep consistently contributed to the sleep quality-connectivity effects found for young adults and multiple sleep components varied in older adults. The results suggest that poor sleep affects the salience networks in both males and females, with variations in adaptive patterns depending on age. The default mode network appears particularly sensitive to sleep impairments in females, consistent with early desegregation of brain networks and increased risk for dementia. The alterations in young adults support the hyperarousal hypothesis as a potential contributor to insomnia.

## Introduction

Sleep constitutes a fundamental aspect of our lives and allows us to function effectively. Sleep patterns vary across individuals due to many factors, including biological sex and the aging process. Sex differences in sleep develop in adolescence and persist throughout adulthood (Campbell et al., 2012). Studies consistently find that women report suffering from worse subjective sleep quality than men, including a greater likelihood of insomnia (Baker et al., 2020; Green et al., 2012; Madrid-Valero, 2017; Zhang & Wing, 2006). Some of these sex differences stem from different sex steroids and different sensitivities to hormonal fluctuations, such as the menstrual cycle (Baker & Driver, 2007; Meers et al., 2019; Moline et al., 2004; Mong & Cumano, 2016; Lord et al., 2014). Other sex differences interact with the aging process, stemming from pregnancy, menopause, and other life transitions (Adan & Natale, 2002; Baker & Driver, 2007; Kervezee et al., 2018; Kravitz et al., 2008; Meers et al., 2019; Shaver et al., 2015; Tonetti et al., 2008; Roenneberg et al., 2007). Across both sexes, obtaining the required amount of sleep becomes more difficult with advanced age (Altena et al., 2010; Neikrug & Ancoli-Israel, 2010). Older adults often experience decreased slow-wave sleep (SWS), rapid-eye movement (REM), and sleep efficiency (Altena et al., 2010; Neikrug & Ancoli-Israel, 2010; Ohayon et al., 2004). Moreover, both intensity and occurrence of sleep disorders increase with aging, including insomnia, sleep-disordered breathing, and REM sleep-behavior disorder (Ancoli-Israel & Cooke, 2005; Neikrug & Ancoli-Israel, 2010; Mong & Cumano, 2016; Zhang & Wing, 2006). These changes in sleep are important because they are associated with a decrease in quality of life (Alapin et al., 2000; Madrid-Valero, 2017; Sella et. al., 2023). Relatedly, declines in sleep quality are associated with cognitive decline, including an increase in mild cognitive impairment (D’Rozario et al., 2020; Mander et al., 2016) and dementia (Kroeger & Vetrivelan, 2023; Mander et al., 2016; Scullin, 2017). Sleep disturbances also can contribute to neurodegeneration due to hypoxia, reduced clearance of metabolites, and increased neuroinflammation, among other important biological risk associated with poor sleep (Kroeger & Vetrivelan, 2023; Xie et al., 2013; Yaffe et al., 2011; Zhu et al., 2012).

However, how sex and age differences in sleep affect brain function and cognition in the context of dementia is less well understood (Alfini et al., 2020). For instance, hormonal changes across aging, particularly in females, may interact with sleep quality in ways that specifically influence cognitive decline risk (Gurvich et al., 2018). Additionally, sex-specific brain network organization and aging trajectories may lead to different vulnerabilities or compensatory mechanisms underlying altered sleep quality. Understanding these complex interactions is crucial for developing targeted prevention and intervention strategies that account for both biological sex and age. Moreover, analysis of cognitive functions in relation to these variables, particularly aging, may reveal potential brain patterns associated with the development of dementia. Although many studies have investigated the relationships between sex, aging, sleep, or brain function, there is little to no investigation on their intersection. Given the central role the brain plays in regulating sleep, hormone cycles, and cognition, the present study investigated associations with brain connectivity and sleep quality across age and sex. The results of our study may hint at potential mechanisms contributing to the differential impact of sleep disruptions on cognitive health. Subsequently, these findings may inform more personalized approaches to preventing or managing cognitive decline and dementia.

Research on self-reported sleep quality in young adults has implicated alterations in the somatomotor network (SMN), salience network (SN), and the amygdala. Among healthy young adults, correlations between sleep quality and resting-state functional connectivity (rsFC) show associations between poorer sleep quality and increased connectivity in brain regions associated with wakefulness (Bai et al., 2022; Fenrico et al., 2022; Klumpp et al., 2018; Zhang et al., 2013). In a large sample of males, poor sleep quality was associated with greater rsFC in sensory and SMN, including the precentral gyrus (PreCG), supplementary motor area (SMA), and postcentral gyrus (PoCG) (Bai et al., 2022). Similarly, higher global PSQI was associated with increased connectivity between the amygdala with the ACC (part of the SN), PoCG, and frontal lobe in young adults with anxiety and depression (Klumpp et al., 2018). These findings are consistent with the hypothesis of insomnia hyperarousal, which posits that high arousal during sleep-onset latency leads to difficulty in falling asleep (Buysee et al., 2011; Riemann et al., 2010). Moreover, as young adults become more sleep deprived, their patterns of rsFC resemble older adults in subcortical and cerebellar brain networks—also linked to movement (Zhou et al., 2017). This latter finding further suggests that, at least networks related to arousal and movement, poor sleep may accelerate the aging process in the brain.

However, studies exploring the relationship between age and and subjective sleep quality on rsFC have mostly implicated alterations in the default mode network (DMN) (Amorim et al., 2018; Federico et al., 2022; Liu et al., 2018). For example, poorer sleep quality in young adults was associated with greater rsFC between the medial temporal lobes (MTL) and both anterior and posterior regions of the default mode network (DMN) in younger adults (Liu et al., 2018). In contrast, older adults did not show any relationships with sleep quality and connectivity between the MTL and DMN (Liu et al., 2018). Importantly, this study did not investigate the extent to which sex may have accounted for some of this variability among either the young or older adult sample. In another study, older adults exhibited greater negative associations between sleep quality and rsFC (i.e., poorer sleep was associated with less connectivity) in networks with central nodes in the occipital cortex, lateral parietal cortex, precuneus, hippocampus, and middle temporal cortex relative to middle-aged adults (Amorim et al., 2018). Other studies have investigated the variability of sleep quality and rsFC among older adult samples. For example, McKinnon et al. (2016) demonstrated decreased DMN-temporal and DMN-parietal rsFC in sleep-disturbed MCI adults compared to sleep-normal MCI adults. Overall, these studies suggest sleep-related alterations within the DMN with advanced age, highlighting how sleep might be one critical factor that explains overall decreases in DMN rsFC as a function of age and dementia found in previous studies (Andrews-Hanna et al., 2007; Spreng & Schacter, 2012; Tomasi & Volkow, 2012; Zhang et al., 2014). The few interactions with age might suggest that younger adults with poor sleep are able to compensate by upregulating within-network connectivity whereas older adults with poor sleep can no longer compensate, thereby leading to decreases in within-network connectivity–a possible sign of more advanced biological aging (for review, see McDonough et al., 2022).

Despite the prevalence of sex differences in sleep (e.g., Ballard et al., 2022; Ballard et al., 2023; Hajali et al., 2019), few studies have investigated how sex may moderate the sleep quality-rsFC relationship and their intersection with age. Generally, males show greater within-network rsFC in networks with core frontal nodes (e.g., executive function, dorsal attention, anterior DMN) than women (Biswal et al., 2010; Ficek-Tania et al., 2023; Zonneveld et al., 2019). Compared to males, females show greater within-network rsFC in the DMN, especially in posterior brain regions such as the parietal cortex (Biswal et al., 2010; Conrin et al., 2018; Ficek-Tania et al., 2023). Interactions between sex and age also have been observed. For example, the greater within-network rsFC in posterior DMN in women decreases with older age to become similar to men’s (Ficek-Tania et al., 2023). This aligns with the finding that sleep duration disparities in men versus women decrease with age (Rahimi-Eichi et al., 2025). Similarly, rsFC between the DMN and somatosensory networks are stronger in women than men in middle-age but not in old age (Zonneveld et al., 2019). These sex differences in middle-age have been attributed to major fluctuations in sex hormones that might put more wear and tear on the DMN (Ficek-Tania et al., 2023). Indeed, studies have shown that the menstrual cycle and oral contraception use alter rsFC in multiple networks, including the DMN (Petersen et al., 2014; Pletzer et al., 2016). Also, among only females, late postmenopausal females showed greater connectivity between the prefrontal cortex and cerebellum relative to females prior to or in early postmenopause (Ballard et al., 2022; Ballard et al., 2023). These group effects were associated with lower sex hormones in late postmenopausal females and better objective sleep quality (as measured by actigraphy). These studies suggest that postmenopausal females in middle age largely exhibit greater rsFC both within and between networks. Such increases in between-network connectivity is found with advanced age and dementia risk, sometimes referred to as network-desegregation (Chan et al., 2014, 2021), thus suggesting that females with more sleep disturbances might biologically age faster than their male counterparts. These female-specific patterns also are consistent with the greater risk for dementia in females than males (Gong et al., 2023).

Investigating early alterations in brain functioning due to sleep impairments holds promise to reveal early mechanisms of cognitive decline and risk for dementia. Given the little work that has investigated both age and sex as moderators of the effects of sleep on rsFC, the present study seeks to fill in this research gap. By leveraging fMRI and self-report questionnaires, we investigated how age and sex moderated the relationship between sleep quality and rsFC. We also explored how components of sleep quality contributed most to any observed effects and whether the observed effects were related to cognition. Understanding early alterations in brain connectivity may aid in our overall understanding of the variable effects of sleep quality in different populations. In addition, our results may inform targeted interventions to address sleep-related issues in older adults while also considering sex-specific effects.

## Methods

### Participants

Study participants (N = 107) were recruited from the Tuscaloosa and Birmingham area in Alabama via medical centers, hospital ads, social media, churches, word of mouth, and existing registries including from the Alabama Research Institute on Aging and UAB’s Comprehensive Center for Healthy Aging from December 2016 to April 2019. All participants were excluded if they had contra-indicators for MRI, were left-handed, had a prior diagnosis of any neurological condition, stroke, traumatic brain injury, claustrophobia, or history of substance abuse. Potential participants were included if they were free of dementia as measured by the St. Louis University Mental Status (Tariq et al., 2006), spoke English fluently, were right-handed, and had at least one risk for dementia. Self-reported dementia risks included subjective memory complaints, less than a high school education, African American or Hispanic ethnoracial category, mild head trauma, family history of AD, current diagnosis of hypertension or systolic blood pressure greater than 140 mmHg, current diagnosis or a family history of heart disease, current diagnosis of high total cholesterol, history or current use of smoking tobacco, current diagnosis or family history of diabetes, and body mass index greater than 30 kg/m2 (Livingston et al., 2017; Perkins et al., 1997). All participants gave informed consent using methods approved by the Institutional Review Board at The University of Alabama. Vision was normal or corrected to normal using MR-compatible glasses or contact lenses. Twelve participants were removed because they did not complete the PSQI responses. Demographic characteristics in the final sample are shown in Table 1.

### Measure of Subjective Sleep Quality

Subjective sleep quality was collected using the PSQI (Buysse et al., 1989). The PSQI is a nineteen-question self-report divided into seven components: subjective sleep quality, sleep latency, sleep duration, habitual sleep efficiency, sleep disturbance, sleep medication, and daytime dysfunction. The PSQI components and global score was used as a continuum, with higher scores correlating with worsening subjective sleep quality. PSQI has high internal consistency (Cronbach’s α = 0.83) and its validity is supported by consistency with sleep diary data (Buysse et al., 1989).

### Dementia Screener

The Saint Louis University Mental Status Exam (SLUMS; Tariq et al., 2006) was used to screen participants for dementia. The assessment consisted of 11 questions that measured a variety of cognitive domains including orientation, memory, attention, and executive functioning. The scores were summed with a maximum value of 30 (see Table 1).

### Measures of Cognition

Participants completed a battery of cognitive tests across four domains: processing speed, executive function, language, and spatial episodic memory. Processing speed was assessed using the Digit Comparison Task (Hedden et al., 2002; Salthouse & Babcock, 1991), Trail Making Test A (Reitan, 1959), and mean response times for correct items from the Attention Network Test (ANT) (Fan et al., 2002). Items were scored such that higher numbers represent better processing speed. Executive function was measured using the Trail Making Test B and the executive attention score from conflict trials relative to the baseline trials in the ANT. Language was assessed using the Wide Range Achievement Test 4 (WRAT-4) (Wilkinson, 2006). These tests assessed word pronunciation to increasingly difficult words and the ability to complete missing words in a sentence to assess comprehension. Spatial episodic memory was measured using the Designs task from the Wechsler Memory Scale IV (Drozdick et al., 2018). In this task, participants viewed abstract lines and a 4 x 4 grid for ten seconds and then had to choose the correct set out of distractors and place them on a blank grid in the correct location. The immediate recall, delayed recall, and delayed recognition tests were all highly correlated with one another and so were averaged together.

### MRI Acquisition

MRI images were collected using a 3T Siemens PRISMA scanner at Birmingham, AL. T1-weighted structural MP2RAGE scans were acquired using (parallel acquisition acceleration type = GRAPPA; acceleration factor = 3, TR = 5000 ms, TE = 2.93 ms, TI 1 = 700 ms, TI 2 = 2030 ms, flip angle 1 = 4°, flip angle 2 = 5°, FOV = 256 mm, matrix = 240 × 256 mm2, in-plane resolution = 1.0 × 1.0 mm2). Two 5-minute resting-state scans were concatenated, totaling 350 volumes. Participants were told to close their eyes but not fall asleep. The functional scans used T2*-weighted EPI sequences (56 interleaved axial slices, 2.5 mm thickness, TR = 1720 ms, TE = 35.8 ms, flip angle = 73°, FOV = 260 mm, matrix = 104 × 104 mm, in-plane resolution = 2.5 × 2.5 mm2, multi-band acceleration factor = 4).

### Preprocessing

Functional and anatomical data were preprocessed using a flexible preprocessing pipeline in the CONN toolbox (Nieto-Castanon, 2020c; Whitfield-Gabrieli & Nieto-Castanon, 2012), including creation of voxel-displacement maps, realignment with susceptibility distortion correction using fieldmaps, outlier detection, indirect segmentation and MNI-space normalization, and smoothing. Functional data were realigned using SPM12 realign & unwarp procedure (Andersson et al., 2001) integrating fieldmaps for susceptibility distortion correction, where all scans were coregistered to a reference image (first scan of the first session) using a least squares approach and a 6 parameter (rigid body) transformation, and resampled using b-spline interpolation (Friston et al., 1995) to simultaneously correct for motion, magnetic susceptibility geometric distortions, and their interaction. Potential outlier scans were identified using ART (Whitfield-Gabrieli et al., 2011) as acquisitions with framewise displacement above 0.5 mm or global BOLD signal changes above 3 standard deviations (Power et al., 2014), and a reference BOLD image was computed for each subject by averaging all scans excluding outliers. Functional and anatomical data were coregistered and normalized into standard MNI space, segmented into gray matter, white matter, and CSF tissue classes, and resampled to 2 mm isotropic voxels following an indirect normalization procedure (Calhoun et al., 2017) using SPM unified segmentation and normalization algorithm (Ashburner, 2007; Ashburner & Friston, 2005) with the default IXI-549 tissue probability map template. Last, functional data were smoothed using spatial convolution with a Gaussian kernel of 8 mm full width half maximum (FWHM).

Functional data were denoised using a standard denoising pipeline (Nieto-Castanon, 2020b), including the regression of potential confounding effects characterized by motion parameters and their first order derivatives (12 factors) (Friston et al., 1996), outlier scans and their first order derivatives (below 122 factors) (Power et al., 2014), and a constant term (1 factor) within each functional run, followed by bandpass frequency filtering of the BOLD timeseries (Hallquist et al., 2013) between 0.008 Hz and 0.09 Hz. From the number of noise terms included in this denoising strategy, the effective degrees of freedom of the BOLD signal after denoising were estimated to range from 26 to 91.4 (average 81.3) across all subjects (Nieto-Castanon, submitted).

## Statistical Analysis

Seed-based connectivity maps (SBC) were estimated characterizing the patterns of functional connectivity with HPC-ICA networks (Nieto-Castanon & Whitfield-Gabrieli, 2022). Functional connectivity strength was represented by Fisher-transformed bivariate correlation coefficients from a weighted general linear model (weighted-GLM (Nieto-Castanon, 2020d), defined separately for each pair of seed and target areas, modeling the association between their BOLD signal timeseries. Seed-to-voxel comparison was conducted using DMN (medial prefrontal cortex [PFC], lateral parietal lobe left [LP], posterior cingulate cortex [PCC]), salience network (anterior cingulate cortex [ACC], anterior insular [Ant. Insula], rostral PFC, supramarginal gyrus [SMG]), and the amygdala. Our independent variables of interest included age, sex, global PSQI score and their interactions. Group-level analyses were performed using a General Linear Model (GLM) (Nieto-Castanon, 2020e). For each individual voxel a separate GLM was estimated, with first-level connectivity measures at this voxel as dependent variables (one independent sample per subject), and groups or other subject-level identifiers as independent variables. Voxel-level hypotheses were evaluated using multivariate parametric statistics with random-effects across subjects and sample covariance estimation across multiple measurements. Inferences were performed at the level of individual clusters (groups of contiguous voxels). Cluster-level inferences were based on parametric statistics from Gaussian Random Field theory (Nieto-Castanon, 2020a; Worsley et al., 1996). Results were thresholded using a combination of a cluster-forming p < 0.001 voxel-level threshold, and a familywise corrected p-FDR < 0.05 cluster-size threshold (Chumbley et al., 2010).

Connectivity values were extracted and further analyzed in R version 4.3.1. When significant interactions occurred within a network, simple slopes were calculated to assess the nature of the interaction, correcting for the number of seeds in that network using Bonferroni correction. Following initial analysis, significant interactions were broken down by PSQI component scores to better understand how sleep quality impacted functional connectivity. Additionally, composite scores of cognition were correlated with age, sex, sleep quality, and functional connectivity in a Pearson correlation matrix.

To assess how the patterns of sleep quality and functional connectivity were associated with cognition, we extracted the significant connectivity values and performed PLS regression analysis in MATLAB (http://www.rotman-baycrest.on.ca/pls). PLS regression was chosen to capture the shared variance between the predictors of interest (age, sex, sleep quality) and functional connectivity that also varied with the four cognitive constructs in the study. Moreover, because a single analysis is conducted, no corrections for multiple comparisons are required. Each of our predictors, their interactions, and the four cognitive constructs formed an Nx11 Y matrix. The values from the significant connectivity pairs formed an Nx6 X matrix. The cross product of the Y-matrix and X-matrix was decomposed into a mutually orthogonal latent variable (LV) using singular value decomposition. The LV scores represented the weights of the brain factors that contributed to correlations with the predictors. The resulting LV was statistically evaluated by 1,000 permutations. A measure of significance was calculated by estimating the proportion of times the permuted singular value was higher than the observed singular value. Bootstrapping was conducted on the LV scores to determine the reliability of each brain metric using 1,000 replicated samples. A bootstrap ratio (BSR) was then calculated by dividing the saliences by the standard error of the generated bootstrap distribution. The bootstrap ratio is approximately equivalent to a z-score, whereby an absolute bootstrap ratio greater than 1.96 corresponds roughly to p < 0.05.

We assessed the robustness and generalizability using three machine learning models (linear regression, robust linear regression, and support vector machine regression). Separately for each model, we used five-fold cross-validation to support the significant interactions found. The relevant interaction formula was entered to train the model. The predicted data was compared to the left-out data to create root mean squared error (RMSE) and mean absolute error (MAE) values. These values were then compared with models in which brain connectivity assignments were randomly shuffled 1,000 times to create a null distribution. If the error from the actual models was lower than the 95% confidence interval from this null distribution, we infer that the interactions can be predicted in a new sample and thus are generalizable. Otherwise, the interactions might be more specific to individuals that more closely represent our sample. A tuning grid was used that varied the cost function with four values (.01, .1, 1, 10) and varied the loss function with two values (L1, L2). The epsilon parameter for support vector regression was set to .05.

## Results

### Default Mode Network

**Figure 1:**
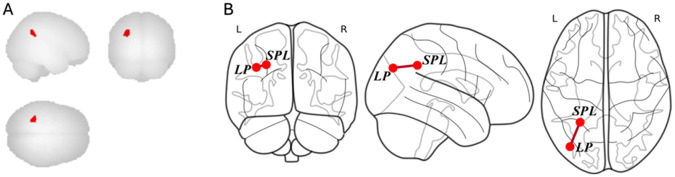
Brain regions showing a significant three-way interaction of age, sex, and global PSQI score on functional connectivity. (A) The significant cluster identified in the left superior parietal lobule (SPL) is displayed on sagittal, coronal, and axial surfaces. (B) Schematic representation of the significant functional connectivity between the left lateral parietal (LP) node of the default mode network and the left SPL. The strength and direction of this connectivity were moderated by age in females, where higher sleep disturbance (PSQI score) was associated with reduced connectivity in younger females but increased connectivity in older females *Notes:* LP, lateral parietal; SPL, superior parietal lobule; L, left; R, right.

We first assessed functional connectivity in the DMN. We found a three-way interaction between age, sex, and global PSQI score on functional connectivity between the DMN nodes and the frontoparietal network (FPN) spanning the left superior parietal lobule (SPL; x = −28, y = −50, z = 36; k = 128; cluster p-FWE = .046, peak p-uncorrected < .000005) (see Fig. 1). No significant main effects or two-way interactions were found.

**Figure 2:**
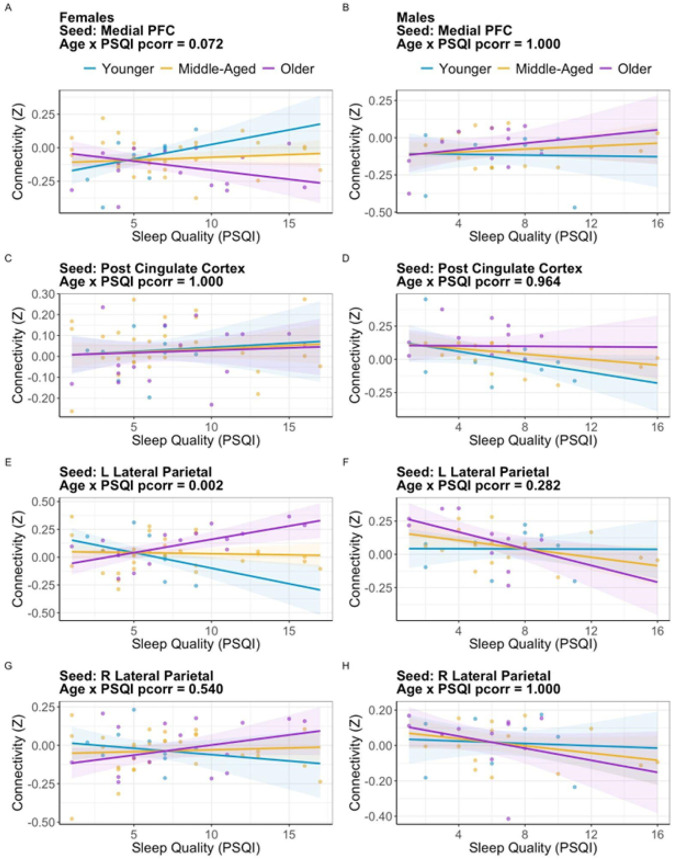
Interaction between age, sex, and sleep quality (PSQI) on functional connectivity between DMN seeds and the left superior parietal lobule (SPL). (A) In females, connectivity from the medial prefrontal cortex (mPFC) showed a trending age × PSQI interaction (pcorr = .072). (B) In males, no significant interaction was observed for the mPFC. (C, D) No significant age × PSQI interactions were found for the posterior cingulate cortex (PCC) in either females or males. (E) In females, connectivity from the left lateral parietal (LP) cortex demonstrated a significant age × PSQI interaction (pcorr = .002). Poorer sleep quality (a higher PSQI score) was associated with reduced connectivity in younger females but was associated with increased connectivity in older females. (F) No corresponding significant interaction was present for the left LP in males. (G, H) The right lateral parietal cortex showed no significant interactions in either sex.

To assess which DMN nodes contributed to this overall connectivity pattern, we calculated the three-way interaction for each node, correcting for multiple comparisons. Of the four nodes in the DMN (medial PFC, PCC, bilateral LP), only left LP reached our threshold of significance (*Beta* = .076, *se* = .019, *pcorr* = .00078). We next calculated two-way age x global PSQI score interactions separately for each sex. In males, age did not moderate the association between PSQI with DMN functional connectivity (*Beta* = −.055, *se* = .030, *pcorr* = .28). For females, age significantly moderated the association between PSQI with DMN functional connectivity (*Beta* = .10, *se* = .027, *pcorr* = .002). Among younger females, higher global PSQI scores were associated with reduced connectivity between the left LP and the left SPL. Among older females, higher global PSQI scores were associated with increased connectivity between the left LP and the left SPL (see Fig. 2).

Lastly, we analyzed the various sleep components that comprised the global PSQI score to better understand what aspects of sleep were associated with this pattern. We found that Component 1 (sleep quality), Component 2 (sleep latency), and Component 3 (sleep duration) were significantly correlated with left LP-SPL connectivity in young females whereas Component 3 and Component 4 (sleep efficiency) were significantly correlated with left LP-SPL in older females.

### Salience Network

**Figure 3:**
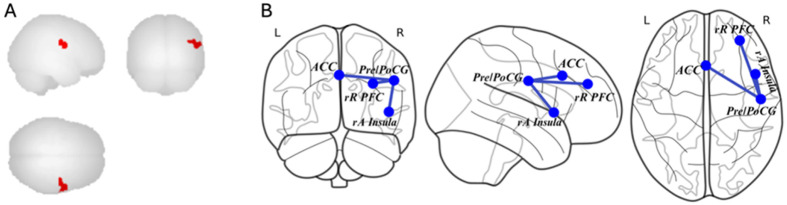
Brain regions showing a significant two-way interaction between age and global PSQI score on functional connectivity within the salience network. **(A)** The significant cluster in the right precentral and postcentral gyrus (Pre/PoCG) is displayed on sagittal, coronal, and axial surfaces). **(B)** Schematic representation of functional connectivity between salience network nodes—including the anterior cingulate cortex (ACC) and bilateral anterior insula (L-aInsula, R-aInsula)—and the right Pre/PoCG. The strength of this connectivity was moderated by age, with increasing age associated with altered connectivity patterns in relation to sleep disturbance.

We next assessed functional connectivity in the SN. We found a two-way interaction between age and global PSQI score on connectivity between the SN nodes and a cluster in the right lateral sensorimotor network spanning the precentral and postcentral gyrus (Pre/PoCG; x = 52, y = −10, z = 30; k = 203; cluster p-FWE = .0019, peak p-uncorrected < .00005)(see Fig. 3).

In young adults, global PSQI scores were positively correlated with connectivity between the Pre/PoCG and 1) the right anterior insula, 2) right rostral PFC, and 3) ACC. In older adults, global PSQI scores were negatively correlated with connectivity between the Pre/PoCG and 1) the right anterior insula, 2) right rostral PFC, and 3) ACC. No significant main effects, other two-way interactions, or three-way interactions were found.

Sleep quality was positively associated with connectivity in younger adults but negatively in older adults for the right anterior insula, right rostral PFC, and ACC. Connectivity between these regions and the Pre/PoCG was further associated with distinct PSQI components across age groups.

When separating the various sleep components of the PSQI, we found many of components were related to SN-Pre/PoCG connectivity in both young and older adults except Component 4 (sleep efficiency) and Component 5 (sleep disturbances) (see Table 3). In younger adults, Component 2 (sleep latency) was the strongest and most reliable across the three significant SN nodes. In older adults, the strongest and most reliable relationships occurred for Components 1, 2, and 3.

### Amygdala

No whole-brain connectivity values were significant for the amygdala seed. Therefore, no follow-up analyses were conducted.

### Robustness Tests

We next assessed robustness and generalizability using machine learning for each of the significant connectivity pairs relative to a null distribution. For the DMN-FPN parietal connectivity (three-way interaction), we found each of the 6 fit metrics were better than chance relative to the null permutation (*p* < .05). For the SN-SMN connectivity, we found 4 were better than chance for the ACC seed, 3 were better than chance for the anterior insula seed, and 6 were better than chance for the rostral PFC seed. Thus, the DMN-FPN and the SN-SMN (rostral PFC) showed reliable robustness and generalizability to held-out samples, whereas the other SN-SMN seeds (anterior cingulate cortex and anterior insula) were not as robust.

### Cognition

Zero-order Pearson correlations can be found in Table 4. Only the DMN-FPN connectivity was correlated with spatial memory (*p* = 0.019). However, this relationship was not significant after controlling for multiple comparisons. PLS-R was used to associate functional connectivity measures with four cognitive constructs: processing speed, executive function, language, and spatial memory. Two latent variables were significant. The first latent variable explained 65.13% of the covariance in the data (p < 0.001). This latent variable was associated with the salience connectivity pattern that varied as a function of age and sleep quality and was not associated with any of the cognitive variables in this study.

The second latent variable explained 20.06% of the covariance in the data (p = 0.040). This latent variable was associated with the default mode connectivity pattern in the left lateral parietal cortex that varied as a function of age, sex, and sleep quality. Greater connectivity between this seed of the default mode network and the left superior parietal lobule was associated with poorer spatial episodic memory.

## Discussion

The present study investigated the moderating effects of age and sex on the relationship between sleep quality and rsFC in the DMN, SN, and amygdala. We found two separate patterns of altered between-network connectivity associated with poorer sleep quality: an age and sex dependent pattern between the DMN and FPN and an agedependent pattern between the SN and SM network. Only DMN connectivity relationships were found to be behaviorally relevant. Below, we discuss the relevance of these patterns to theories of hyperarousal and neural desegregation.

### Age and Sex Moderate Sleep Quality and Functional Connectivity in the DMN

Relationships between sleep quality and functional connectivity in the DMN were moderated by both age and sex. Specifically, in younger females, poorer sleep quality was associated with lower connectivity between the left LP (DMN) and left SPL connectivity (FPN), whereas in older females, poorer sleep quality was associated with greater connectivity. No relationship was found in males. A recent study showed that females exhibited greater alterations in functional connectivity in posterior nodes of the DMN relative to men (Ficek-Tani et al., 2023), hinting at a selective vulnerability for women in this network. The increase in connectivity observed in older females with poor sleep quality also resembles hyperconnectivity patterns observed in preclinical Alzheimer's disease (AD) (McDonough & Allen, 2019; Mormino et al., 2011; Sheline et al., 2010). This aberrant functional connectivity is consistent with a meta-analysis showing risks for AD converge on brain function in the lateral parietal cortex of the FPN and medial parietal cortex in the DMN (McDonough et al., 2020).

Differences in the direction of the effects between age groups might suggest different underlying causes. For example, SPL connectivity differs between sleep deprivation and sleep disorders (Javaheripour et al., 2019; Li et al., 2014). Specifically, lower SPL activation was found in sleep deprived subjects while higher SPL activation was found in insomnia patients compared to controls (Javaheripour et al., 2019; Li et al., 2014). Thus, the age differences might be due to different types of sleep problems: poor quality sleep in young females and insomnia in older females. This suggestion is consistent with the PSQI component sores with unique associations with sleep quality and latency in young females, but poor sleep efficiency uniquely in older females.

We also observed that these connectivity differences were isolated to the left hemisphere. Previous studies have shown that sleep is characterized by right hemispheric dominance in EEG power, at least in right-handed individuals (Ferrara et al., 2002; Park & Shin, 2017), which aligns with the Homeostatic Hypothesis. This hypothesis states that the left hemisphere requires more recuperation during sleep than the right hemisphere. Thus, sleep-related disturbances might be more sensitive to left hemispheric functioning, at least between the DMN and FPN. This interpretation is strengthened by Liu et al. (2018), who obtained similar left-lateralized effects associated with sleep disruption in older adults. Interestingly, the aforementioned meta-analysis localized the aberrant lateral parietal brain activity with AD risk to the left hemisphere as well (McDonough et al., 2020), consistent with a link between sleep deficits and AD.

Breaking down sleep quality into its separate components revealed that the DMN-FPN connectivity was associated with sleep quality, latency, and duration in younger females. Only sleep duration and efficiency significantly correlated with that connectivity pattern in older females. While numerous studies have investigated the relationship between PSQI global score and functional connectivity, few have considered each PSQI component’s contribution to connectivity. Although no studies we are aware of have discovered PSQI component interaction with DMN or FPN rsFC, Sung et al. (2020) found increased sleep disturbance (component 5) correlated with decreased DMN gray matter volume. Meanwhile, Killgore et al. (2023) found objective measures of sleep latency was negatively correlated with DMN connectivity to other cortical areas while sleep duration and sleep efficiency were positively correlated. Li et al. (2021) found that longer sleep duration correlated with lower cognitive scores, enforcing the role of sleep in cognitive function. Nevertheless, extrapolation between these studies and our present study is difficult due to different sleep quality and neuroimaging measurements and lack of overlapping PSQI components.

Together, these age differences suggest different underlying causes for network-related sleep disruptions and different approaches for intervention. Past research supports the role of psychological resilience as a mediator between DMN connectivity and sleep quality, although samples only included younger adults (Chen et al., 2023; Shi et al., 2022; Zhang et al., 2022). Intervention for younger females may be aimed at increasing sleep hygiene associated with increasing sleep quality, decreasing latency, and extending duration to prevent altered connectivity. By addressing these aspects early in the lifespan, disruptions in functional brain networks might be prevented. In contrast, intervention for older females may include maintaining brain health through proper diet and physical activity to reduce insomnia and increase sleep duration (Key & Szabo-Reed, 2023).

### Age Moderates Sleep Quality and Functional Connectivity across Both Sexes

Across both sexes, age moderated the connectivity of the SN with the Pre/PoCG (part of the SMN). These patterns were found for multiple seeds in the salience network, albeit mostly right lateralized (right anterior insula, right rostral PFC, and ACC). Given the different patterns in the SN compared with the DMN (in terms of sex, laterality, and direction), we suspect that the mechanisms are quite different. Rather than being related to AD risk, these patterns are consistent with previous research showing hyperconnectivity in the SN and SMN associated with poor sleep quality in the context of the hyperarousal hypothesis, at least in younger adults (Chen et al., 2014; Cheng et al., 2022; Khazaie et al., 2017; Li et al., 2022; Yin et al., 2024). Hyperconnectivity in the SN and SMN has been assumed to increase external stimulus sensitivity, making it difficult to fall asleep and stay asleep (Cheng et al., 2022; Li et al., 2022). In contrast, poor sleep quality in older adults correlated with decreased connectivity between these regions. This pattern is inconsistent with the idea that older adults also have hyperarousal that impairs sleep quality. Interestingly, recent work suggests that older adults with sleep disturbances and high beta-amyloid accumulation also exhibit increased *within*-network connectivity in older adults relative to those with low beta-amyloid (Kim et al., 2024). However, that study did not investigate between-network connectivity as found in the present study. The right-lateralization of our findings may speak to the right hemi-aging model wherein decline in right hemisphere function is touted to be more marked with aging (Dolcos et al., 2002), indicating a possible new mechanism (i.e., poor sleep) for this hypothesis.

Breaking down sleep quality into its separate components revealed that the SN-SMN connectivity was related to sleep quality, latency, duration, medications, and daytime dysfunction in both younger and older adults. This suggests that more severe or widespread sleep disruptions must occur before alterations occur for SN-SMN connectivity. Similar results were seen in Bai et al. (2022), who found a positive correlation between sleep latency and DMN-SMN connectivity in young males. Given that older adults showed the opposite relationships, these differences could indicate different mechanisms (and neural effects) of sleep problems in young and older adults. Alternatively, this decrease in connectivity among older adults is *inconsistent* with the hyperarousal model and may represent different pharmacological effect on SN. In older adults, sleep medications may operate on the brain by reducing hyperarousal mechanisms but may not sufficiently improve sleep quality, thus leading both to increased sleep medication use and continued sleep problems. In young adults, the medications may work more effectively or might be the result of different types of sleep medications. Among the people who have sleep problems, older adults used sleep medication more than young adults. More research needs to be conducted to better link type of sleep medications on SN connectivity.

### Absence of Amygdala Connectivity Effects

Contrary to our hypotheses, we found no significant correlation between sleep quality and amygdala connectivity. This negative result is surprising considering past studies that have demonstrated links between different sleep disturbance forms and altered amygdala function (Goldstein & Walker, 2014; Huang et al., 2012). Various reasons could account for our findings. First, the effect of sleep quality on amygdala connectivity may be subtler or context-dependent when compared to effects seen within the DMN and salience networks. Second, the characteristics of our sample or our methodological approach may have differed from previous studies, such as the type of correction for multiple comparisons to detect amygdala-related effects. A third consideration is that some factors like stress or emotional reactivity might mediate the relationship between sleep quality and amygdala connectivity that were not included in our analyses.

### Cognitive Correlates of Functional Connectivity Patterns

To assess the behavioral relevance of sleep quality and connectivity findings, we investigated the relationship with these patterns and our cognitive battery. Although multiple relationships were found when conducting zero-order correlations, the most robust relationship found using PLS-R was for the DMN-FP relationship and spatial episodic memory. Specifically, the patterns of hyperconnectivity we found in older adults are associated with poorer spatial episodic memory. This pattern is consistent with prior findings of greater network desegregation association with poorer episodic memory (Chan et al., 2014) and offers support for the long-established view that the parietal lobe is involved in processing and integrating spatial information (Javaheripour et al., 2019; Sack, 2009; Wagner et al., 2005). Spatial processing, including spatial navigation, and episodic memory are particularly impaired in preclinical stages of AD (Backman et al., 2004; Ohman et al., 2021). Furthermore, women are more significantly impacted by Alzheimer’s than men, both in prevalence and severity (Gong et al., 2023; Laws et al., 2018). Our study showed that older women experience significant increased DMN-FP connectivity. Together, these relationships strengthen the relevance of these findings to brain vulnerabilities specific to women and the risk for dementia.

### Limitations and Future Directions

The current study utilized self-report measures of sleep quality. Future research should include objective measures like polysomnography or other physiological assessments of sleep quality. Second, the cross-sectional nature of our study precludes our ability to make clear causal inferences. We do not know whether age and/or sex differences in functional connectivity causes poor sleep quality (as suggested by the hyperarousal hypothesis) or whether poor sleep quality causes aberrant functional connectivity. Longitudinal studies would clarify whether changes in sleep quality occurred first or after alterations in brain connectivity and intervention studies can better test whether normalizing functional connectivity to a youthlike state might also improve sleep quality. Future investigations could also contribute to understanding the mechanisms by which sleep quality interacts with brain connectivity and cognition. For example, outstanding questions include whether the effects of poor sleep on brain function might operate through neuroinflammation, oxidative stress, or glymphatic clearance. Knowing how sleep disturbance interacts with disease mechanisms may lead to novel sleep related therapeutic and preventive measures.

## Conclusion

The present study illustrates the effect of age and sex in moderating how sleep quality affects between-network functional connectivity during rest. The patterns found suggest that older women might be particularly vulnerable to network-related differences due to sleep problems that accelerate brain aging and episodic memory decline. Our current findings suggest several important clinical implications, including the consideration of age and sex. The differences in patterns for males and females and between younger and older adults indicate that sleep problems might require intervention by demographic group. Second, our data suggest that addressing sleep latency in young females may be particularly important because younger females demonstrated a significant relationship between sleep latency with both DMN-FPN and SN-SMN connectivity, indicating that latency may disrupt multiple brain networks. In support of this, cognitive-behavioral therapy for insomnia (CBT-I) has been shown to be more beneficial for younger females than other groups (Trauer et al., 2015). Third, given the evidence that the alteration in DMN connectivity contributes to a decline in spatial memory performance, managing sleep disorders could lead to improved spatial functioning via the DMN. This evidence is consistent with studies mounting evidence of sleep intervention improvement in cognition in healthy aging and mild cognitive impairment (Mander et al., 2016).

## Figures and Tables

**Figure 4:**
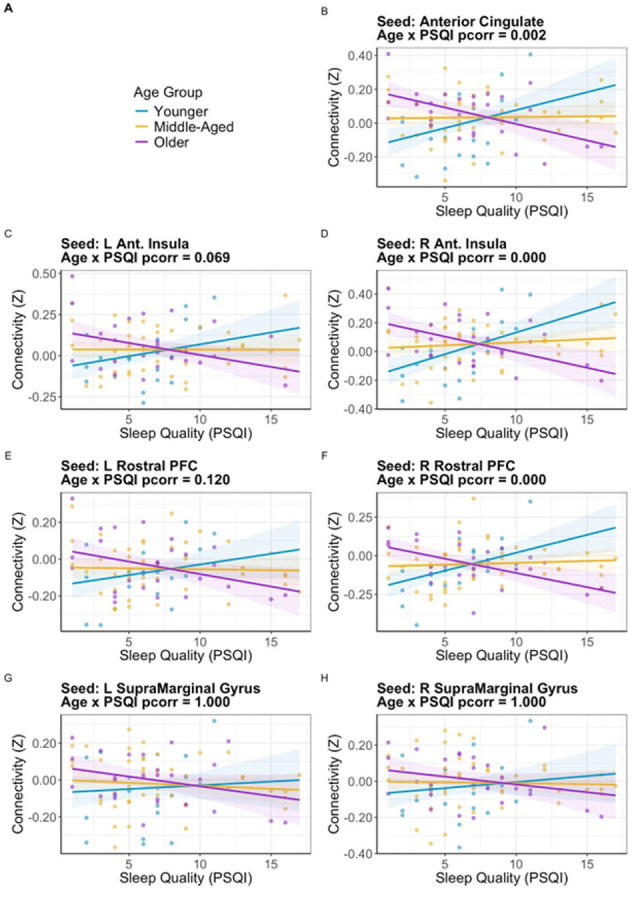
Interaction between age group and sleep quality (PSQI) on functional connectivity between salience network seeds and the right precentral/postcentral gyrus (Pre/PoCG). **(A)**Legend showing color-coded age groups: younger, middle-aged, and older adults. **(B)**Connectivity from the anterior cingulate cortex (ACC) showed a significant age × PSQI interaction, with a positive correlation in younger adults and a negative correlation in older adults. **(C)** Left anterior insula showed a trending interaction (pcorr = .069), while **(D)** right anterior insula showed a significant interaction, following the same age-related pattern. **(E, F)**Right rostral PFC also demonstrated a significant age × PSQI interaction, whereas the left rostral PFC did not. **(G, H)** No significant effects were observed for the left or right supramarginal gyrus Sleep quality was positively associated with connectivity in younger adults but negatively in older adults for the right anterior insula, right rostral PFC, and ACC. Connectivity between these regions and the Pre/PoCG was further associated with distinct PSQI components across age groups.

**Figure 5:**
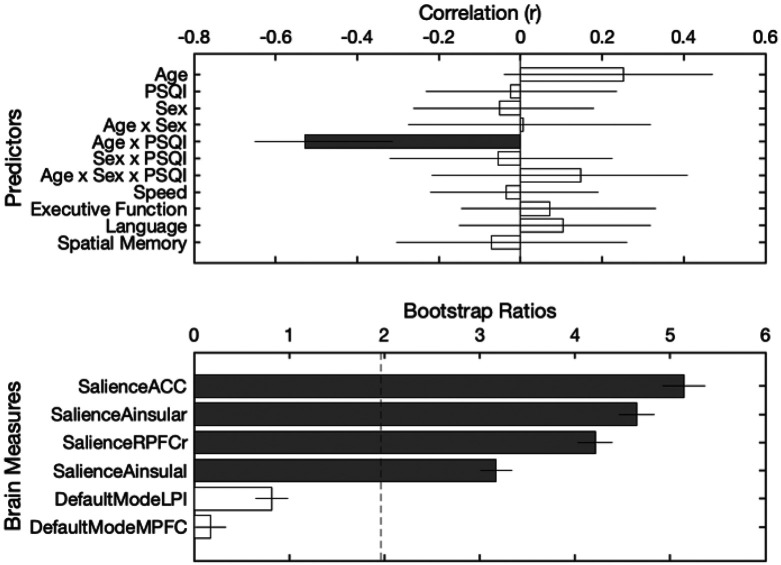
Partial least squares regression (PLS-R) results linking brain connectivity patterns to age, sleep, and cognitive variables. This latent variable explained 65.13% of the covariance in the data (p < .001), capturing a salience network connectivity pattern modulated by age and sleep quality but not associated with any cognitive performance outcomes.

**Figure 6:**
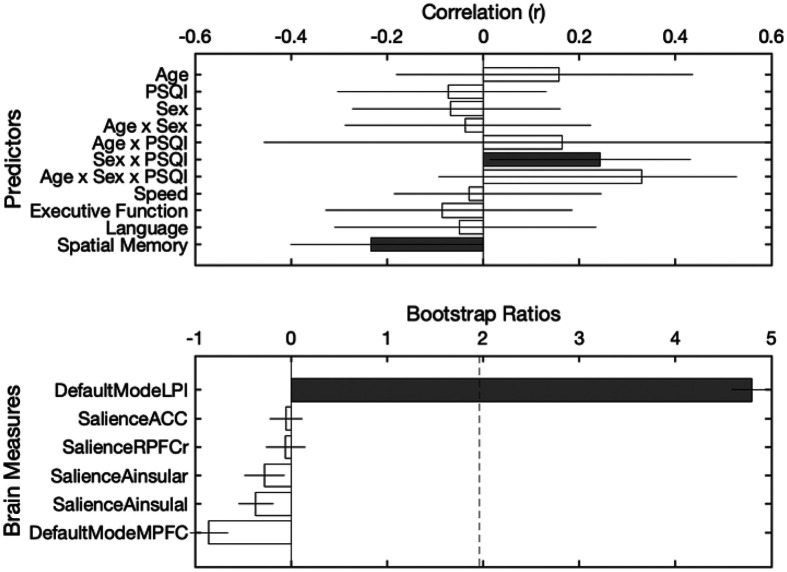
Partial least squares regression (PLS-R) results identifying brain–behavior associations involving default mode network connectivity. This latent variable explained 20.06% of the covariance in the data (p = .040) and was associated with a default mode connectivity pattern modulated by age, sex, and sleep quality. Notably, higher DMN–LP connectivity was associated with poorer spatial episodic memory performance.

**Figure 7:**
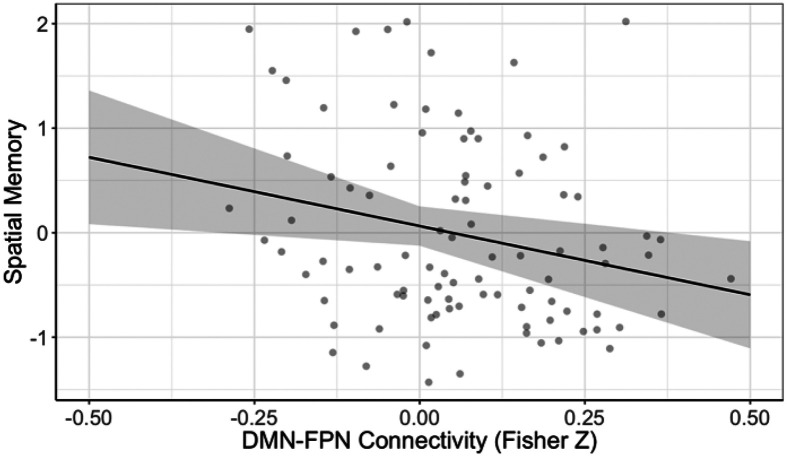
Association between DMN–FPN functional connectivity and spatial memory performance.

**Table 1:** Demographics of Study Participants

Factor	Mean/N	SD/%
Age	52.81	16.35
Sex		
Male	36	37.90%
Female	59	62.10%
Race		
White	56	58.95%
Black	30	31.58%
Other	9	9.47%
Education (Years)^a^	14.62	2.41
PSQI	6.84	3.88
SLUMS^b^	26.43	2.66

Notes:

a Education was missing for 8 participants

b SLUMS was not given to young adults

**Table 2:** Brain Regions Showing Significant Connectivity

	Cluster	Center Coordinate			
Seed	Size	x	y	z	Z
Default Mode Network					
Total	128	−28	−50	+ 36	3.097
Left Superior Parietal Lobe	28	−28	−52	+ 40	
Left Posterior Supramarginal Gyrus	4	−32	−48	+ 38	
Salience Network					
Total	203	+ 52	−10	+ 30	4.0
Right Precentral Gyrus	100	+ 58	−2	+ 26	
Right Postcentral Gyrus	82	+ 56	−2	+ 24	

**Table 3:** Correlations Between Network Connectivity and PSQI Sleep Components by Age and Sex

		PSQI Component						
Covariates	PSQI Total	1 Quality	2 Latency	3 Duration	4 Efficiency	5 Disturbances	6 Medications	7 Dysfunction
Younger Female								
DMN: L Lateral Parietal	−.29**	−.27*	−.22*	−.33**	.055	−.11	.0026	−.14
Older Female								
DMN: L Lateral Parietal	.32**	.17	.17	.39***	.27**	.16	008	−.067
Younger Adult								
SN: ACC	.30**	.19	.35***	.13	−.088	−.0096	.33**	.13
SN: R Ant. Insula	.38***	.21*	.36***	.33***	.057	.027	.15	.22*
SN: R Rostral PFC	.35***	.22*	.39***	.093	.070	−.092	.28**	.24*
Older Adult								
SN: ACC	−.33***	−.21*	−.28**	−.25*	−.12	−.16	−.23*	−.12
SN: R Ant. Insula	−.34***	−.24*	−.21*	−.24*	−.055	−.20	−.18	−.24*
SN: R Rostral PFC	−.34***	−.30**	−.24*	−.26*	−.015	−.091	−.21*	−.25*

*** p < .001

** p < .01

* p < .05

**Table 4:** Zero-Order Pearson Correlations Between Age, Sleep Quality, Network Connectivity, and Cognitive Performance

	Age	PSQI	SN: ACC	SN: Right Ant. Insula	SN: Right Rostral PFC	DMN: Left Lateral Parietal	Spatial Memory	Speed	Executive Function	Language
Age	-	.02	.26	.21	.11	.20	−.66	−.59	−.40	.05
PSQI		-	−.06	.02	−.01	−.07	.05	−.10	.01	−.30
SN - ACC	**		-	.60	.45	.04	−.09	.01	.17	.09
SN - Right Ant. Insula	*		***		.41	−.10	.00	−.09	.02	.11
SN - Right Rostral PFC			***	***	-	.09	−.11	−.07	−.05	−.04
DMN - Left Lateral Parietal						-	−.24	−.05	−.11	−.04
Spatial Memory	***					*	-	.65	.52	.28
Speed	***						***	-	.63	.30
Executive Function	***						***	***	-	.23
Language		**						**	*	-

*** p < .001

** p < .01

* p < .05

**Table 5:** P-values from Permutation Tests for Machine Learning Model Robustness

	Linear Regression		Robust Linear Regression		Support Vector Machine	
Seed	RMSE	MAE	RMSE	MAE	RMSE	MAE
DMN-Lateral Parietal	< .001	.002	.002	.003	.002	.003
SN-Anterior Cingulate Cortex	.001	.037	.059	.407	< .001	.001
SN-Anterior Insula	.014	.052	.033	.157	.009	.057
SN-rostral PFC	.001	.041	<.001	.003	.001	.008

*Notes.* DMN = Default Mode Network, SN = Salience Network, RMSE = root mean square error, MAE = mean absolute error.

## Data Availability

The datasets generated and analyzed during the current study are not publicly available due the fact that they constitute an excerpt of research in progress but are available from the corresponding author on reasonable request.
